# Comparison of 1-year neurological outcome between intra-hospital and extra-hospital cardiac arrest survivors submitted to mild therapeutic hypothermia in a community-based setting in Brazil

**DOI:** 10.1186/cc12246

**Published:** 2013-03-19

**Authors:** CA Abreu Filho, A Andrade, A Neto, S Santos, M Bracco, E Silva, A Baruzzi, J Teixeira

**Affiliations:** 1 Hospital Israelita Albert Einstein, São Paulo, Brazil; 2 Hospital Municipal Dr. Moysés Deutsch, São Paulo, Brazil

## Introduction

Mild therapeutic hypothermia (MTH) is the most powerful therapy to improve survival and neurologic outcome after out-of-hospital cardiac arrest. Such benefit may also occur for unconscious patients after in-hospital cardiac arrest. The aim is to compare 1-year evolution of neurological outcomes of patients treated with MTH after in-hospital versus out-of-hospital cardiac arrest.

## Methods

A prospective study of patients treated with MTH after cardiac arrest in a community hospital in São Paulo, Brazil. After return of spontaneous circulation, unconscious survivors received MTH using topical ice and cold saline infusions in order to achieve a 32 to 34°C goal temperature, within 6 hours of cardiac arrest, and maintained for 24 hours. Esophageal temperature was monitored; continuous intravenous sedation-analgesia was maintained for 48 hours after initiation of MTH. The Glasgow Outcome Scale (GOS) was used to analyze the neurological outcomes after hospital discharge.

## Results

From January 2009 to April 2012, 84 patients submitted to MTH were divided into two groups: Group 1, 54 patients presented out-of-hospital cardiac arrest; and Group 2, 30 patients had intra-hospital cardiac arrest. Both groups were similar regarding to gender; Group 2 tended to be older (mean age 44.3 years vs. 33.5 years, *P *= 0.07), and had more frequently asystole as the cardiac arrest rhythm (45% vs. 15 %, *P *= 0.10). Group 2 had shorter duration of resuscitation (12.3 minutes vs. 33.7 minutes, *P *= 0.03), longer time to hypothermia initiation (309.3 minutes vs. 212.8 minutes, *P *= 0.04), longer hospital stay after cardiac arrest (50.7 days vs. 32.4 days, *P *= 0.01) and worse neurological outcome, characterized by the presence of GOS ≤3 at 30 days (68.4% vs. 35.1%, *P *= 0.03). Hospital mortality was 5.5% in Group 1 and 13.3% in Group 2 (*P *= 0.21). The 1-year survival rate was 85.1% in Group 1 and 83.3% in Group 2 (*P *= 0.43); after 13year follow-up, GOS ≤3 was present in 30.4% of Group 2 patients and in 11.1% of Group 1 patients (*P *= 0.04).

## Conclusion

Midterm neurological outcome of MTH after in hospital cardiac arrest seems to be not as good as after out-of-hospital cardiac arrest. Delay in hypothermia initiation, older age and associated comorbidities could explain the worse evolution of this group of patients.

